# Complement C3 and C4, but not their regulators or activated products, are associated with incident metabolic syndrome: the CODAM study

**DOI:** 10.1007/s12020-018-1712-3

**Published:** 2018-08-21

**Authors:** Ying Xin, Elisabeth Hertle, Carla J. H. van der Kallen, Casper G. Schalkwijk, Coen D. A. Stehouwer, Marleen M. J. van Greevenbroek

**Affiliations:** Department of Internal Medicine, Maastricht University Medical Centre and CARIM School for Cardiovascular Diseases, Maastricht University, Maastricht, The Netherlands

**Keywords:** Human, Complement activation, Metabolic syndrome, Incidence, Longitudinal

## Abstract

**Purpose:**

We investigated the associations of components of the alternative (C3, C3a, Bb, factor D [FD], factor H [FH], properdin) and the classical complement pathway (C4, C1q, C1-inhibitor [C1-INH]) with prevalent and incident metabolic syndrome in a cohort with a moderately increased risk of cardiometabolic disease.

**Methods:**

The study cohort was comprised of 574 participants (61% men, age 59.6 ± 7.0 years) at baseline and 489 participants after 7-year follow-up. Multiple logistic regression analyses were done to investigate the associations of concentrations of baseline plasma complement (standardized values) with prevalent and incident (in those without metabolic syndrome at baseline, *n* = 189) metabolic syndrome.

**Results:**

C3 (odds ratio (OR) = 1.48 [95% confidence interval: 1.02; 2.14]) and C4 (OR = 1.95 [1.32; 2.88]), but none of the other complement components were associated with incident metabolic syndrome (*n* = 40 cases). Notably, in the cross-sectional analyses, we did observe higher levels of C3a (OR = 1.25 [1.03; 1.52]), FH (OR = 2.93 [2.24; 3.83]), and properdin (OR = 1.88 [1.50; 2.34]), in addition to C3 (OR = 3.60 [2.73; 4.75]) and C4 (OR = 1.39 [1.13; 1.69]), in those with the metabolic syndrome compared to those without, while no association was observed for FD, Bb, C1q, or C1-INH.

**Conclusions:**

In the cross-sectional analyses, the effects sizes (standardized regression coefficients) for C3 and C4 were similar to those of (some of) the regulators and activators, yet only C3 and C4 were associated with incident disease. These findings suggest a role for C3 and C4, but not their regulators or activated products, in the development of the metabolic syndrome.

## Introduction

The metabolic syndrome is a cluster of cardiovascular risk factors that is characterized by central obesity, dyslipidemia, hypertension, and insulin resistance. Obesity is one of the initial events in the pathological processes that define the metabolic syndrome [1]. Dysfunctional adipose tissue is considered an important driver in the development of the adverse metabolic profiles in people with obesity [1].

The complement system is an essential part of the innate immune system. It is widely expressed in adipose tissue, and its expression is increased in adipose tissue of obese individuals. The complement system has three main activation pathways: the alternative, the classical, and the lectin pathway (as reviewed in [2], Supplementary Fig. S1). Previous research has shown that the expression of some complement components, especially of the alternative and classical pathways, is higher in adipose tissue of people with obesity (as reviewed in [3]). Several, mainly cross-sectional, studies have addressed the associations of individual complement factors with adverse metabolic profiles (as reviewed in [4]). However, these studies often focused on one or a few complement components and on a specific aspect of metabolism. At the same time, studies on the associations of complement with metabolic diseases mostly focused on advanced disease states such as CVD (as reviewed in [5]) or T2DM [6, 7]. As it stands, the association of complement with the metabolic syndrome, which is the main comorbidity of obesity and predisposes to both CVD and T2DM [8], has received much less attention.

Most available studies on complement and the metabolic syndrome are cross-sectional and mainly focused on C3 [9–11], the central component of the alternative pathway, and its degradation product C3a-desarg (also known as acylation-stimulating protein, ASP) [12, 13]. To the best of our knowledge, the prospective association of C3 with the metabolic syndrome was only reported in two human observational studies. One demonstrated that Caucasian individuals in highest C3 quartile had a higher risk to develop the metabolic syndrome during 3 years of follow-up [14]. The other study was performed in Chinese men and reported a similar positive association for C3. In this latter study, C4, the downstream component of the classical (and lectin) pathway, was also reported to be positively associated with the development of the metabolic syndrome [15]. The information on the relationships, especially the prospective ones, of other complement proteins, regulators, and activated products with the metabolic syndrome is even more limited, particularly in humans. Factor D [FD], also known as adipsin, is the rate-limiting enzyme of alternative pathway and is mainly produced by adipocytes. Although previous data revealed a possible role for FD in lipid metabolism and β cell function [16, 17], little is known about its association with the metabolic syndrome. Factor B [FB], factor H [FH], and properdin are important components and regulators of the alternative complement pathway. Thus far, only one cross-sectional study in Caucasian men reported positive associations of FH and FB with several aspects of metabolism [18]. Although a possible role of properdin in lipid metabolism was recently demonstrated in a properdin-deficient mouse model [19], human data are still lacking. In addition, Hillian et al. revealed a protective effect of C1q, the initiator of classical pathway activation, on high-fat diet-induced hepatic insulin resistance and impaired glucose homeostasis in a C1q-knockout mouse model. And apolipoprotein E-deficient mice on an atherogenic diet that were treated with C1-inhibitor [C1-INH] showed a decrease in serum triglyceride (TG) [20]. Yet again, except for some information on C4, human data on the association of the classical pathway with the metabolic syndrome are largely lacking.

In the present study, we hypothesized that activation of the alternative and the classical complement pathways, represented by higher circulating levels of their components, is associated with the metabolic syndrome. Therefore, we investigated in a Caucasian cohort with moderately increased risk of cardiometabolic disease, the associations of these components, especially those of the alternative (i.e., C3, C3a, Bb, FD, FH, and properdin) and classical (i.e., C1q, C1-INH, and C4) pathways with the prevalence of the metabolic syndrome, as well as its incidence during a 7-year follow-up period.

## Material and methods

### Participants

The Cohort on Diabetes and Atherosclerosis Maastricht (CODAM) is a prospective observational cohort. Participants were selected from a large population-based study as described previously [21]. CODAM participants are of Caucasian descent and >40 years of age with one or more of the following characteristics: body mass index (BMI) >25 kg/m^2^; use of antihypertensive medication; positive family history of T2DM; postprandial blood glucose level >6.0 mmol/L; history of gestational diabetes and/or glycosuria. Data were collected for 574 participants at baseline and for 495 participants at follow-up. The median follow-up time was 7 years (interquartile range (IQR) 6.9–7.1). After exclusion of participants who had missing data (*n* = 37, 6%), 537 individuals with complete data were included in the cross-sectional analyses. Similarly, of the 209 participants who did not have the metabolic syndrome at baseline, 20 (10%) had either missing information or were lost to follow-up and were therefore not included in the prospective analyses. A flowchart for the inclusion of the participants is shown in Fig. S2. This study was approved by the medical ethics committee of Maastricht University. All participants gave written informed consent.

### Measurements

#### Complement measurements

Participants were asked to stop their lipid-modifying medication 14 days and any other medication 1 day prior to the measurements. Blood samples were collected after an overnight fast and stored at –80 °C until use. FH and C1-INH were measured in EDTA plasma using commercially available ELISA kits (FH: DuoSet, R&D Systems, Minneapolis, MN, USA; C1-INH: MicroVue C1-INH EIA kit, Quidel, Catalog no. A037, San Diego, USA) according to the manufacturer’s instructions, inter-assay variation was 13.5% and 4.4%, respectively. C4 was measured in serum by auto-analyzer (Hitachi) using a Roche kit assay (Roche Diagnostics Netherland B.V., Almere, The Netherlands). The inter-assay variation was 2.0%. Measurements of other complement factors (i.e., C3, C3a, Bb, FD, properdin, and C1q) were performed as previously described [22–24].

#### Definition of the metabolic syndrome

The metabolic syndrome was defined according to the AHA/NHLBI & IDF 2009 harmonized criteria [25]. Participants who met three or more of the following criteria were classified as having the metabolic syndrome: (1) waist circumference (waist) ≥88 cm in men and ≥102 cm in women; (2) TG ≥1.7 mmol/L, and/or use of medication for elevated TG; (3) high-density lipoprotein cholesterol (HDL) <1.0 mmol/L in men, and <1.3 mmol/L in women, and/or use of medication for reduced HDL; (4) systolic blood pressure (SBP) ≥130 mmHg and/or diastolic blood pressure (DBP) ≥85 mmHg, and/or use of antihypertensive medication; (5) fasting glucose ≥100 mg/dl, and/or use of glucose-lowering medication.

#### Other variables

Anthropometric measurements, plasma biochemistry, lifestyle factors, and medication use were obtained exactly as described in our previous publications [21, 22, 26, 27].

### Statistical analyses

Variables are presented as mean (SD) or percentages. Those with skewed distribution are presented as median (IQR). Independent sample *t*-test, Mann–Whitney U test, and chi-square test were used to compare two groups, as indicated.

Variables with skewed distribution (C3a, TG, fasting plasma glucose, HOMA2-IR, aspartate aminotransferase [AST], alanine aminotransferase [ALT], gamma-glutamyl transferase [GGT], C-reactive protein [CRP], serum amyloid A [SAA], interleukin-6 [IL-6], interleukin-8 [IL-8], tumor necrosis factor-a [TNF-a], and soluble intercellular adhesion molecule-1 [sICAM1]) were log_2_ transformed prior further analyses. For the various complement components standardized values ([individual’s observed values−population mean]/standard deviation of the population) were calculated to allow direct comparison of their effect sizes in the regression analyses. All analyses were performed using IBM SPSS statistics version 22 and a two-tailed *P*-value of <0.05 was considered significant.

#### Cross-sectional analyses

At baseline, multiple logistic regression analyses were done to investigate the associations of plasma complement concentrations (standardized values, main independent variable) with the prevalence of the metabolic syndrome (main outcome). Subsequently, multiple linear regression analyses were performed to investigate the associations between complement concentrations and individual components of the metabolic syndrome (i.e., TG, HDL, fasting plasma glucose, SBP, DBP, and waist circumference). All analyses were initially adjusted for age (years) and sex (male/female) (Model 1), then additionally for medication use (lipid-modifying, antihypertensive, and/or glucose-lowering, each yes/no), smoking status (current or previous tobacco smoking, yes/no), alcohol consumption (g/d), physical activity (METs/week), and energy intake (kJ/d) to control for potential confounding (Model 2).

#### Prospective analyses

In those who did not have the metabolic syndrome at baseline, multiple logistic regression analyses were done to evaluate the associations between baseline complement concentrations and the incident metabolic syndrome (main outcome). Models and adjustments were as mentioned for the cross-sectional analyses.

#### Sensitivity analyses

Use of medication to control blood pressure, plasma glucose, and dyslipidemia could affect an individual’s risk to develop the metabolic syndrome during follow-up. For this reason, we repeated the prospective analyses while excluding all participants who used lipid-modifying, antihypertensive, and/or glucose-lowering medication at baseline and/or at follow-up. Besides, plasma complement concentrations may be affected by certain disease conditions. The prospective analyses were therefore also repeated after excluding participants who, at baseline, had (1) acute or chronic infections (CRP >10 mg/L), (2) a (suspected) history of autoimmune disease (defined as self-reported current chronic joint inflammation/rheumatoid arthritis or a severe intestinal disorder that lasted for the past 3 months or longer), (3) a self-reported current malignant condition or cancer, or (4) self-reported liver disease.

#### Additional analyses

Several additional analyses were done to explore the possible pathways that may contribute to the associations between complement and incidence of the metabolic syndrome. First, participants who developed the metabolic syndrome during follow-up may at baseline, even though they did not meet the criteria of having the metabolic syndrome, have already had a somewhat worse metabolic status. Thus, to find out via which of the metabolic syndrome components the complement system may contribute to development of the metabolic syndrome, we additionally adjusted the prospective analyses for the baseline levels of the individual components of the metabolic syndrome. Besides, higher systemic complement levels may reflect ongoing liver dysfunction [27], which may accelerate the development of the metabolic syndrome [28]. Therefore, additional adjustments were done for liver function markers (i.e., AST, ALT, and GGT). Also, the metabolic syndrome may coincide with an enhanced acute phase response (APR) [29] and complement C3 and C4 are known as acute phase proteins [30]. Prolonged APR results in a state of chronic low-grade inflammation (LGI) which may contribute to the pathogenesis of metabolic syndrome (as reviewed in [31]). Therefore, the analyses were adjusted for several markers of LGI, i.e., CRP, SAA, IL-6, IL-8, TNF-a, and sICAM1. Lastly, since complement activation may induce insulin resistance [32], which is an important contributor to the metabolic syndrome, the prospective regression analyses were additionally adjusted for baseline levels of HOMA2-IR.

## Results

### Baseline characteristics of the study population

At baseline, complete data were available for 537 participants of whom 328 (61%) had the metabolic syndrome (Table 1). Participants with the metabolic syndrome were more often men, were slightly older, had lower insulin sensitivity, and worse LGI, had higher prevalence of CVD, were more likely to use medication, and reported lower physical activity. In addition, the plasma concentrations of several complement factors (i.e., C3, C3a, FD, FH, properdin, C4) were higher in participants with the metabolic syndrome.

**Table 1:** Baseline characteristics of the study population according to the presence or absence of the metabolic syndrome at baseline or at follow-up

	Prevalent MetS		Incident MetS	
	MetS at baseline	No MetS at baseline^a^	No MetS at baseline^a^	
			MetS at follow-up	No MetS at follow-up
	*n* = 328 (61%)	*n* = 209	*n* = 40 (21%)	*n* = 149
Age (years)	60.0 ± 6.6	58.4 ± 7.4*	58.2 ± 7.3	58.4 ± 7.4
Sex (% men)	65.5	54.5*	55.0	53.7
BMI (kg/m^2^)	30.0 ± 4.3	26.4 ± 3.6*	27.5 ± 3.6	26.1 ± 3.3^†^
Waist (cm)	104.0 ± 10.8	92.2 ± 10.4*	95.0 ± 9.6	91.3 ± 10.2^†^
Triglycerides (mmol/L)	1.80 (1.30–2.20)	1.00 (0.80–1.40)*	1.35 (1.03–1.50)	1.00 (0.80–1.20)^†^
HDL cholesterol (mmol/L)	1.05 ± 0.27	1.41 ± 0.34*	1.29 ± 0.27	1.45 ± 0.36^†^
Systolic BP (mmHg)	144.8 ± 17.0	131.8 ± 18.4*	136.7 ± 20.1	129.8 ± 16.6^†^
Diastolic BP (mmHg)	83.9 ± 8.9	78.0 ± 8.2*	79.4 ± 8.6	77.4 ± 7.9
Fasting plasma glucose (mmol/L)	6.03 (5.59–7.19)	5.20 (4.97–5.45)*	5.29 (4.94–5.44)	5.19 (4.98–5.45)
Type 2 diabetes (%)	38.7	4.3*	12.5	1.3^†^
Cardiovascular disease (%)	34.5	18.2*	22.5	17.4
HOMA2-IR^b^	2.15 (1.46–3.29)	1.12 (0.84–1.51)*	1.16 (0.88; 1.61)	1.07 (0.85; 1.44)
Inflammation score^c^	0.20 ± 0.96	-0.32 ± 0.98*	−0.19 ± 1.02	−0.47 ± 0.88
Ever smoking (%)	23.5	21.1	10.0	21.5
Alcohol intake (g/d)	7.85 (0.64–21.4)	9.92 (2.30–23.91)	11.23 (2.46–22.51)	8.29 (2.30–23.15)
Physical activity (10^3^ METs/week)	6.37 ± 3.88	7.13 ± 4.49*	7.18 ± 4.99	7.20 ± 4.39
Energy intake (10^3^ kJ/day)	9.27 ± 2.76	9.29 ± 2.80	9.43 ± 2.95	9.29 ± 2.81
Glucose-lowering medication (%)	21.3	1.4*	5.0	0.7
Lipid-modifying medication (%)	23.5	13.4*	22.5	12.1
Antihypertensive medication (%)	48.8	23.4*	35.0	20.1 ^†^
C3 (g/L)	1.07 ± 0.15	0.92 ± 0.13*	0.96 ± 0.12	0.91 ± 0.13^†^
C3a (μg/L)	61.1 (51.1–75.4)	56.3 (47.4–67.6)*	58.5 (47.4–74.2)	55.1 (46.6–67.0)
Bb (mg/L)	0.71 ± 0.18	0.73 ± 0.20	0.71 ± 0.19	0.72 ± 0.20
Factor D (mg/L)	1.02 ± 0.24	0.97 ± 0.23*	0.96 ± 0.26	0.97 ± 0.22
Factor H (mg/L)	350.2 ± 77.7	285.8 ± 60.9*	280.8 ± 52.6	284.3 ± 61.7
Properdin (mg/L)	6.30 ± 1.29	5.63 ± 1.14*	5.70 ± 0.90	5.65 ± 1.18
C1q (mg/L)	73.1 ± 15.6	70.8 ± 14.8	71.5 ± 14.7	71.2 ± 14.8
C1-INH (mg/L)	170.2 ± 12.3	168.6 ± 12.4	170.4 ± 12.0	167.2 ± 12.4
C4 (g/L)	0.30 ± 0.07	0.28 ± 0.07*	0.31 ± 0.07	0.26 ± 0.06^†^

*MetS* metabolic syndrome, *BP* blood pressure

^a^Of those who did not have the metabolic syndrome at baseline (*n* = 209), *n* = 20 were lost to follow-up or had missing information on one or more components of the metabolic syndrome; the total number of participants available for analyses on incident metabolic syndrome is *n* = 189

^b^HOMA2-IR: *n* = 12 missing at baseline, *n* = 1 missing at follow-up

^c^Higher value means more low-grade inflammation

^*^P < 0.05 between participants with and without metabolic syndrome at baseline

^†^*P* < 0.05 between participants who did or not develop metabolic syndrome during the 7-year follow-up period

Of the 189 participants who did not have the metabolic syndrome at baseline, 40 (21%) developed the metabolic syndrome during the 7-year follow-up period. The baseline characteristics of those who did and did not develop the metabolic syndrome during follow-up are also presented separately in Table 1. Already at baseline, those who developed the metabolic syndrome were more obese, had higher TG and lower HDL, higher SBP, higher prevalence of T2DM, and were more likely to use antihypertensive medication. They also had higher concentrations of plasma C3 and C4.

### Cross-sectional associations of proteins, regulators, and activated products of the complement system with prevalence of the metabolic syndrome

All participants who had complete data at baseline (*n* = 537) were included in the cross-sectional analyses. Several components of the alternative pathway (i.e., C3, C3a, FH, and properdin) as well as for one component of the classical pathway activation (C4) were significantly and positively associated with the metabolic syndrome. These associations remained significant in the fully adjusted model (Table 2, Model 2, C3, odds ratio (OR) = 3.60 [95% confidence interval (CI): 2.73; 4.75]; C3a, OR = 1.25 [1.03; 1.52]; FH, OR = 2.93 [2.24; 3.83]; properdin, OR = 1.88 [1.50; 2.34]; C4, OR = 1.39 [95% CI 1.13; 1.69]). The OR of 3.6 for C3 implies that, after adjustment for confounders, those participants who had a 1 SD higher C3 concentration were 3.6 times more likely to have metabolic syndrome. Factor Bb, FD, C1q, and C1-INH were not significantly associated with the metabolic syndrome (Table 2, Model 2, Bb, OR = 0.90 [0.74; 1.09]; FD, OR = 1.06 [0.87; 1.31]; C1q, OR = 1.10 [0.91; 1.34]; C1-INH OR = 1.12 [0.91; 1.36]). In line with this, most complement factors (except for factor Bb) were significantly, and in an adverse direction, associated with one or more individual components of the metabolic syndrome (Supplementary Table S1).

**Table 2:** Cross-sectional associations of complement proteins, regulators, and activated products with prevalence of the metabolic syndrome

	Metabolic syndrome *n* = 537; 328 prevalent cases (61%)			
	Model 1		Model 2	
	OR (95% CI)	*P*-value	OR (95% CI)	*P*-value
C3 (SD)	3.71 [2.86; 4.82]	<0.001	3.60 [2.73; 4.75]	<0.001
C3a (SD)	1.31 [1.09; 1.58]	0.004	1.25 [1.03; 1.52]	0.023
Bb (SD)	0.89 [0.75; 1.07]	0.209	0.90 [0.74; 1.09]	0.279
Factor D (SD)	1.11 [0.92; 1.33]	0.293	1.06 [0.87; 1.31]	0.558
Factor H (SD)	3.00 [2.34; 3.86]	<0.001	2.93 [2.24; 3.83]	<0.001
Properdin (SD)	1.79 [1.46; 2.19]	<0.001	1.88 [1.50; 2.34]	<0.001
C1q (SD)	1.17 [0.98; 1.40]	0.083	1.10 [0.91; 1.34]	0.316
C1-INH (SD)	1.14 [0.95; 1.36]	0.172	1.12 [0.91; 1.36]	0.282
C4 (SD)	1.43 [1.18; 1.72]	<0.001	1.39 [1.13; 1.69]	0.002

All complement components were standardized, C3a was log_2_-transformed prior to standardization

Model 1 (M1) is adjusted for age and sex

Model 2: M1 + medication use (lipid-modifying, antihypertensive, and/or glucose-lowering), smoking status, alcohol consumption, physical activity, and energy intake

### Prospective associations of proteins, regulators, and activated products of the complement system with incidence of the metabolic syndrome

In contrast to the strong associations that were observed in the cross-sectional analyses, only a limited number of complement components were associated with incidence of the metabolic syndrome (Table 3). In the fully adjusted models (Model 2), significant associations were observed for C3 (OR = 1.48 [1.02; 2.14]) and C4 (OR = 1.95 [1.32; 2.88]). Thus, participants with 1 SD higher baseline concentration of C3 or C4 were approximately 1.5 and 2.0 times more likely to develop the metabolic syndrome during the 7-year follow-up period. Positive associations were also observed for C3a and C1-INH, but these did not reach statistical significance (C3a, OR = 1.37 [0.96; 1.96]; C1-INH, OR = 1.34 [0.89; 2.02]). The associations of the other complement factors were weaker and non-significant, with ORs around 1 (Bb, OR = 0.94 [0.64; 1.38]; FD, OR = 0.91 [0.62; 1.34]; FH, OR = 0.87 [0.59; 1.27]; properdin, OR = 1.09 [0.75; 1.58]; C1q, OR = 1.01 [0.69; 1.46], Table 3).

**Table 3:** Prospective associations of baseline concentrations of complement proteins, regulators, and activated products with incidence of the metabolic syndrome

	Incident metabolic syndrome in those who did not have the metabolic syndrome at baseline *n* = 189, 40 incident cases (21%)			
	Model 1		Model 2	
	OR (95% CI)	*P-*value	OR (95% CI)	*P-*value
C3 (SD)	1.55 [1.09; 2.21]	0.016	1.48 [1.02; 2.14]	0.038
C3a (SD)	1.36 [0.97; 1.91]	0.075	1.37 [0.96; 1.96]	0.087
Bb (SD)	0.94 [0.66; 1.34]	0.731	0.94 [0.64; 1.38]	0.749
Factor D (SD)	0.93 [0.64; 1.36]	0.715	0.91 [0.62; 1.34]	0.632
Factor H (SD)	0.95 [0.66; 1.35]	0.760	0.87 [0.59; 1.27]	0.462
Properdin (SD)	1.04 [0.73; 1.47]	0.849	1.09 [0.75; 1.58]	0.662
C1q (SD)	1.02 [0.72; 1.45]	0.907	1.01 [0.69; 1.46]	0.980
C1-INH (SD)	1.36 [0.92; 2.00]	0.122	1.34 [0.89; 2.02]	0.158
C4 (SD)	1.94 [1.34; 2.82]	<0.001	1.95 [1.32; 2.88]	0.001

All complement components were standardized, C3a was log_2_-transformed prior to standardization

Model 1 (M1) is adjusted for age and sex

Model 2: M1 + medication use (lipid-modifying, antihypertensive, and/or glucose-lowering), smoking status, alcohol consumption, physical activity, and energy intake

We evaluated the robustness of the associations of C3 and C4 with development of the metabolic syndrome in several sensitivity analyses. Exclusion of participants who used medication at baseline and/or at follow-up resulted in a substantial decrease in the number of events (*n* = 12), but the results remained largely consistent with the main analyses (Table S2). Likewise, when patients with acute or chronic infections, with a (suspected) history of autoimmune disease, with a current malignant condition/cancer, or with self-reported liver disease were excluded from the analyses, the results remained similar to what was observed in the main analyses (Table S2).

Several additional analyses were done to evaluate how complement may contribute to the development of the metabolic syndrome. For this, first we adjusted the prospective regression analyses for baseline levels of the individuals components of the metabolic syndrome. This substantially attenuated the association of baseline C3, but not C4, with incident metabolic syndrome (C3, OR = 1.04 [0.66; 1.64], C4, OR = 1.73 [1.14; 2.64] Table 4). Second, the development of the metabolic syndrome may also be accelerated by more generalized underlying pathologies such as fatty liver disease, LGI, or insulin resistance. Therefore, we also performed additional adjustments with measurements that reflect fatty liver disease, LGI, and insulin resistance, respectively. Additional adjustment for baseline levels of ALT, AST, and GGT resulted in a slight attenuation of the association of baseline C3 with incidence of the metabolic syndrome, which lost significance (OR = 1.30 [0.87; 1.93]), while for C4 the association remained significant (OR = 2.01 [1.34; 3.00], Table 5). Additional adjustment for LGI had only minor effects on the association of baseline C3 and C4 with incident metabolic syndrome (C3, OR = 1.36 [0.90; 2.05]; C4, OR = 1.91 [1.28; 2.83]), although the association for C3 was, again, no longer significant (Table 5). Also when HOMA2-IR was included, the associations with incident metabolic syndrome were largely consistent with the main results (C3, OR = 1.46 [0.97; 2.21]; C4, OR = 1.94 [1.31; 2.86], Table 5).

**Table 4:** Prospective associations of the baseline concentrations of complement C3 and C4 with incident metabolic syndrome: additional adjustment for baseline levels of the individual components of the metabolic syndrome

	Outcome: incident metabolic syndrome, *n* = 189, 40 cases (21%)			
	C3		C4	
Model	OR (95% CI)	*P-*value	OR (95% CI)	*P-*value
1	1.48 [1.02; 2.14]	0.038	1.95 [1.32; 2.88]	0.001
2a	1.34 [0.90; 2.01]	0.151	1.95 [1.31; 2.89]	0.001
2b	1.27 [0.86; 1.89]	0.244	1.82 [1.23; 2.70]	0.003
2c	1.33 [0.90; 1.95]	0.156	1.72 [1.16; 2.56]	0.007
2d	1.45 [0.99; 2.11]	0.056	1.95 [1.31; 2.90]	0.001
2e	1.48 [1.02; 2.14]	0.038	1.97 [1.33; 2.93]	0.001
2f	1.04 [0.66; 1.64]	0.860	1.73 [1.14; 2.64]	0.011

Model 1 (M1) is adjusted for age, sex, medication use (lipid-modifying, antihypertensive, and/or glucose-lowering), smoking status, alcohol consumption, physical activity, and energy intake

Model 2a: M1 + waist

Model 2b: M1 + triglyceride levels

Model 2c: M1 + HDL cholesterol level

Model 2d: M1 + systolic and diastolic blood pressure levels

Model 2e: M1 + fasting plasma glucose level

Model 2f: M1 + waist, triglyceride, HDL cholesterol, systolic and diastolic blood pressure, and fasting plasma glucose

**Table 5:** Additional analyses: prospective associations of the baseline concentrations of complement C3 and C4 with incident metabolic syndrome adjusted for liver function markers, markers of low-grade inflammation, or insulin-resistance (HOMA2IR)

	Outcome: incident metabolic syndrome *n* = 186, 39 incident cases (22%)			
	C3		C4	
Model	OR (95% CI)	*P*-value	OR (95% CI)	*P-*value
1	1.48 [1.02; 2.15]	0.039	1.96 [1.33; 2.90]	0.001
2a	1.30 [0.87; 1.94]	0.196	2.01 [1.34; 3.00]	0.001
2b	1.36 [0.90; 2.05]	0.139	1.91 [1.28; 2.83]	0.001
2c	1.45 [0.95; 2.20]	0.084	1.93 [1.30; 2.85]	0.001

Model 1 (M1) is adjusted for age, sex, medication use (lipid-modifying, antihypertensive, and/or glucose-lowering), smoking status, alcohol consumption, physical activity, and energy intake

Model 2a: M1 + aspartate aminotransferase, alanine aminotransferase, and gamma-glutamyl transferase levels at baseline, compiled into an average *Z-*score (calculation of average *Z-*score: standardized values for each marker were calculated ([individual’s observed values−population mean]/standard deviation of the population) within each participant, the standardized values of the individual markers were summed, then averaged and the standardized values of this averaged score was included in the regression models)

Model 2b: M1 + C-reactive protein, serum amyloid A, interleukin-6, interleukin-8, tumor necrosis factor-a, and soluble intercellular adhesion molecule-1 levels at baseline, compiled into an average *Z-*score (calculation of average *Z-*score: standardized values for each marker were calculated ([individual’s observed values−population mean]/standard deviation of the population) within each participant, the standardized values of the individual markers were summed, then averaged and the standardized values of this averaged score was included in the regression models)

Model 2c: M1 + HOMA2-IR at baseline

## Discussion

We here show that at baseline, C3 and C4, but also systemic concentrations of C3a, FH, and properdin, were higher in individuals with metabolic syndrome compared to those without. We also show that baseline concentrations of complement C3 and C4 were positively and significantly associated with development of the metabolic syndrome during the 7-year follow-up period. In contrast, this was not the case for the other complement components (i.e., C3a, Bb, FD, FH, properdin, C1q, and C1-INH).

Cross-sectional associations of complement with components of the metabolic syndrome, such as obesity [33] and insulin resistance [34], were described more than 10 years ago. Cross-sectional associations of complement components, mainly C3 and its downstream product C3adesarg/ASP, with the metabolic syndrome were also reported previously [9–13]. In line with this, we confirm the positive associations of C3 and C3a (the precursor of ASP) with prevalence of the metabolic syndrome. Also, we extend previous findings by reporting the associations of factor Bb, FD, FH, and properdin (components and regulators of the alternative pathway), with presence of the metabolic syndrome (significant only for FH and properdin). In our current evaluations, the associations of the proximal factors of the classical pathway, (C1q and C1-INH), with the metabolic syndrome were modest. Only C4 was significantly associated with the presence of the metabolic syndrome. Positive associations between C4 and prevalence of the metabolic syndrome were reported previously [12, 15], although not consistently [11]. Thus, our cross-sectional observations indeed suggested that activation of the complement system, especially the alternative pathway, is related to the metabolic syndrome.

We also evaluated to what extent the components of the complement system were associated with incidence of the metabolic syndrome over a 7-year follow-up period. Positive associations were observed for C3 and C3a, but only reached statistical significance for C3. Notably, the associations of all the other components of the alternative pathway, including FH and properdin, were non-significant with ORs close to 1. This strongly suggests that, despite the observed strong cross-sectional associations, FH and properdin are not risk factors for the development of metabolic syndrome. Two previous human studies have reported on the role of C3 in the development of the metabolic syndrome [14, 15], and their results are in line with our current observation. We additionally observed a very robust association of C4, but not the proximal components of the classical pathway, with development of the metabolic syndrome. To the best of our knowledge, only one previous study reported on the association between C4 and incident metabolic syndrome [15]. In that study, a positive association with incidence of the metabolic syndrome was shown but no adjustments were made for components of the metabolic syndrome. So taken together, in our prospective cohort, systemic concentration of the major complement components C3 and C4, but not their activators or activated products, were associated with incident metabolic syndrome.

The association with incidence of the metabolic syndrome was stronger for C4 than C3. Moreover, for C4, but not for C3, this association was independent of baseline levels of the individual components of the metabolic syndrome and its main underlying metabolic aberrancies. This suggests that the effects of C3 and C4 on the development of the metabolic syndrome are distinct. This is for instance illustrated by the observation that the association of complement C3, but not of C4, with incident metabolic syndrome was partly explained by liver function. Also, activation of the C3–C3a–C3adesArg/ASP axis may provide a partial explanation for the observation that C3 is a risk factor for the development of the metabolic syndrome. Experimental data showed that C3adesArg/ASP stimulates TG synthesis and glucose uptake and inhibits hormone-sensitive lipase in several cell types (as reviewed in [4]). These known effects of C3 and its activation products may affect lipid metabolism and thus contribute to the development of the metabolic syndrome. An exciting novel mechanism of complement activation is the so-called intracellular complement system. Intracellular complement activation was first identified in T cells and has been implicated in the regulation of increased glycolysis and oxidative phosphorylation in Th1 cells [35]. In the circulation, C3 can be spontaneously activated and transformed into C3(H_2_O), the hydrolytic product of C3, via the so-called tick-over mechanism. And recently it was established that many cell types can take up C3(H_2_O) from plasma, and part of this intracellular C3(H_2_O) provides an intracellular source of C3a via a process that is independent of C3 convertase [36]. This latter finding is particularly interesting given our current observation that C3, but not FH or properdin, was associated with the development of the metabolic syndrome. The effects of intracellular C3 activation have not yet been established. However, given the effects of intracellular complement activation on TH1 metabolism, intracellular C3 activation will likely affect metabolism in many cell types and, as such, potentially affect the metabolic dysregulation that characterizes the metabolic syndrome. Notably, C4 cannot be spontaneously activated via the tick-over mechanism, which illustrates that the above-mentioned intracellular route may be particularly relevant for C3. This may add to the possibility that C3 and C4 contribute to the metabolic syndrome via distinct mechanisms.

The available information on C4 in relation to metabolism is limited. Genetic variation in the C4 binding protein (C4BP), which is an inhibitor of the classical and the lectin pathway, has been related to higher blood pressure and higher fasting blood glucose [37]. C4BP may also have a protective effect on β cell function [38]. In addition, recent developments showed that C4a, which is generated upon activation of C4, can bind to and activate the G-coupled protein receptor protease-activated receptor (PAR)1 and PAR4 [39]. Notably, this receptor cannot be activated by C3a [39]. Activation of PAR1 and 4 by C4a leads to cellular activation and enhanced endothelial permeability [39], and PAR1 has been previously implicated in, e.g., cardiac remodeling [40] and hepatic injury [43]. Notably, C4a cannot signal via the C3aR [41]. These data, again, support the notion that in addition to their well-known conjunction on the terminal complement pathway, activation of C3 and C4 may indeed have distinct effects on metabolism. This also opens the path toward the investigation of a possible relation of C4, activated via the classical or the lectin pathway, with platelet activation and/or endothelial dysfunction, irrespective of the extent of terminal pathway activation.

The major strength of our study is that it provides information on several components and regulators of the alternative and the classical complement pathways, measured within one cohort, in the relation to the metabolic syndrome. Another key strength is that it provides prospective information on the development of the metabolic syndrome. Nevertheless, some limitations need to be acknowledged. The number of cases in the prospective analyses was relatively small, which may have limited our power to detect associations when effect sizes were smaller than those of C3 and C4. This may have been the case for C3a and perhaps also C1-INH. With respect to the interpretation of the data, we are limited by the fact that all complement measures were obtained in plasma which does not provide insight in local activation of these complement components relevant metabolic organs. Also, our cohort consists of individuals with a moderately increased risk to develop cardiometabolic diseases, which limits the generalizability of our findings. In addition, our study may be limited by the missing of information for several participants in the cross-sectional (6%) and the prospective (10%) analyses. However, our current observations are substantiated by their consistency with previous reports. Moreover, given our observational study design, we cannot draw causal conclusions on the relationship between complement and the metabolic syndrome. However, our findings are consistent with previous reports and are also supported by suggestive biological evidence, such as the altered metabolic profiles in several complement gene-deficient mouse models [17, 19, 42], which suggests that complement may play a role in the development of metabolic syndrome.

In conclusion, we herein report that complement C3 and C4 are positively associated with incidence of the metabolic syndrome. For C3, but not for C4, these effects appear to be related to the metabolic profile at baseline. Importantly, properdin and FH, which showed strong associations in the cross-sectional analyses, were not related to incident metabolic syndrome. In the discussion, we refer to novel developments in our understanding of activation and signaling of complement in order to provide some explanation for these divergent observations (see graphical summary in Fig. S3), but further work is needed to better understand the etiological role of complement activation in the cellular and metabolic pathways that underlie the development of the metabolic syndrome and related cardiometabolic diseases. Future work should include direct metabolic effects of C3 and C4 as well as effects of their activation products, C3a/C3a-desarg and C4a.

## Electronic supplementary material

Supplementary Information
