# Supplementary material for: Complement C3 and C4, but not their regulators or activated products, are associated with incident metabolic syndrome: the CODAM study
Source: Endocrine. 2018 Aug 21;62(3):617–27. doi: 10.1007/s12020-018-1712-3 (PMC6244913; doi:10.1007/s12020-018-1712-3)
Supplement: Supplementary file 1 — Supplementary Information [file 12020_2018_1712_MOESM1_ESM.docx]

**Supplementary Information for Endocrine:**

**Complement C3 and C4, but not their regulators or activated products, are associated with incident Metabolic Syndrome: the CODAM study**

Ying Xin, Elisabeth Hertle, Carla J. H. van der Kallen, Casper G. Schalkwijk, Coen D. A. Stehouwer, Marleen M. J. van Greevenbroek.

Dept. of Internal Medicine, Maastricht University Medical Centre and CARIM School for Cardiovascular Diseases, Maastricht University, The Netherlands.

**Correspondence:** Dr. Marleen M. J. van Greevenbroek, Dept. of Internal Medicine, Maastricht University Medical Centre and CARIM School for Cardiovascular Diseases, Maastricht University, Universiteitssingel 50, PO Box 616, 6200 MD Maastricht, The Netherlands.

E-mail: [m.vangreevenbroek@maastrichtuniversity.nl](mailto:m.vangreevenbroek@maastrichtuniversity.nl)

**Contents:**

**Figure S1. Activation of the complement system with focus on components that are most relevant for the current study.**

**Figure S2. Flowchart of the individuals included in the main analyses in this study.**

**Figure S3. Graphic summary of the possible explanations for the main findings.**

**Table S1. Cross-sectional associations of complement with components of the metabolic syndrome.**

**Table S2. Sensitivity analyses: Prospective associations of baseline concentrations of complement proteins, regulators and activated products with incident metabolic syndrome.**

**
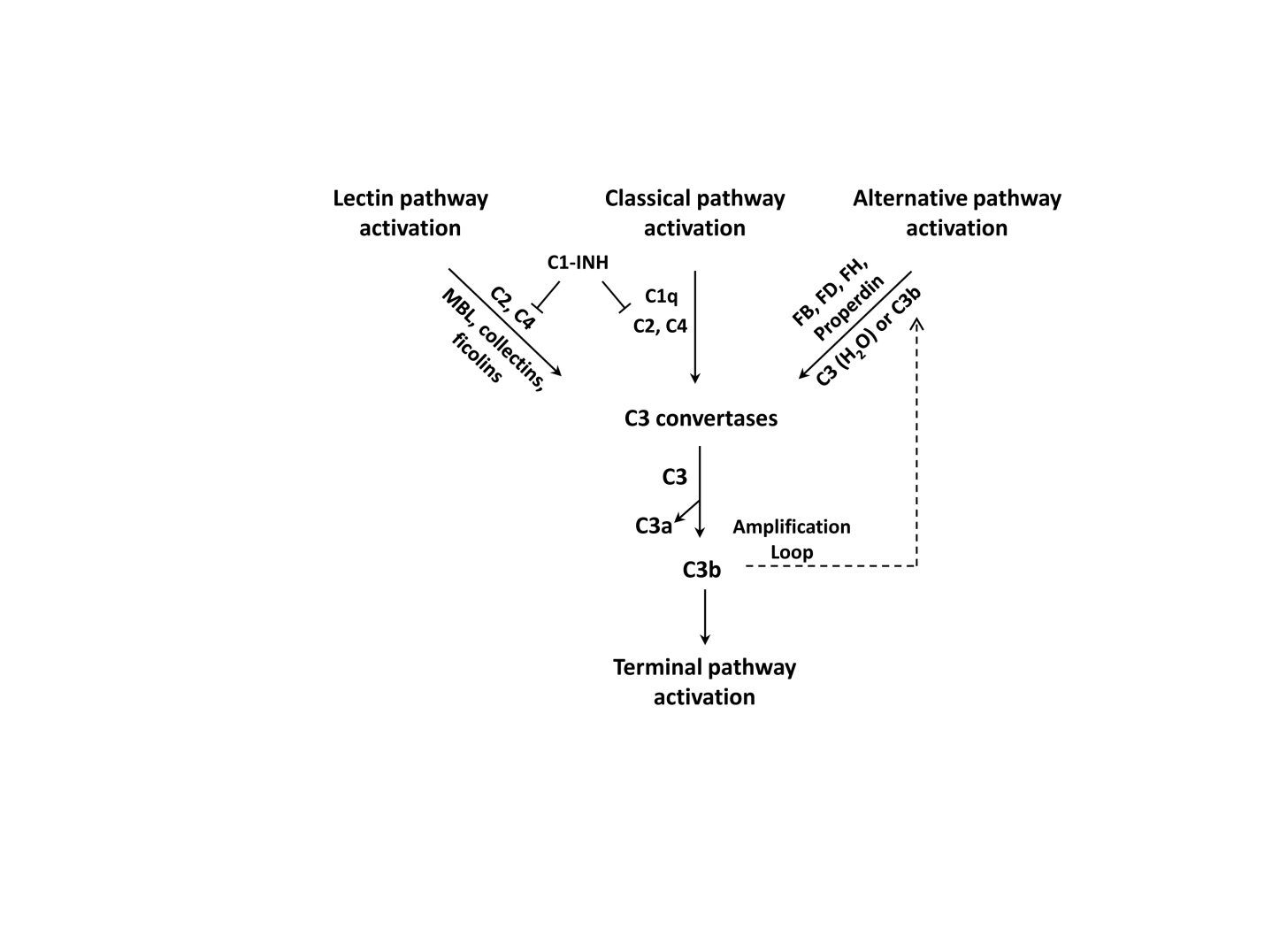
**

**Figure S1. Activation of the complement system with focus on components that are most relevant for the current study.** Complement activation can be initiated via the classical, lectin, or alternative pathway. Classical pathway activation starts with interaction between C1q and its ligands, lectin pathway activation starts with the recognition of its ligands by mannose binding lectin (MBL), collectins, or ficolins. Both can result in the cleavage of C2 and C4 to form C3 convertase (C4b2a). Activation of these two pathways is controlled by the regulator C1-inhibitor (C1INH). The alternative pathway can be initiated via two routes. Activation can start with spontaneous hydrolysis of C3 which generates C3(H_2_O) or with C3b that is generated by the classical or lectin pathways. C3b and C3(H_2_O) make factor B (FB) available for cleavage by factor D (FD). This yields Bb which combines with C3b to generate the alternative pathway C3 convertase (C3bBb). The alternative pathway functions as an amplification loop for all activation pathways. Activation of the alternative pathway is under strict control of several regulatory proteins, including factor H (FH), which acts as a destabilizer of the C3 convertase, and properdin which is a C3 convertase stabilizer. C3 convertases generated via either pathway cleave C3 into C3a and C3b. The anaphylatoxin C3a is rapidly degraded to C3a-desarg, also called acylation stimulating protein (ASP). C3b can contribute to the amplification loop to produce more C3 convertases and, by combining with the C3 convertase, generate a C5 convertase and lead to the activation of the terminal pathway.


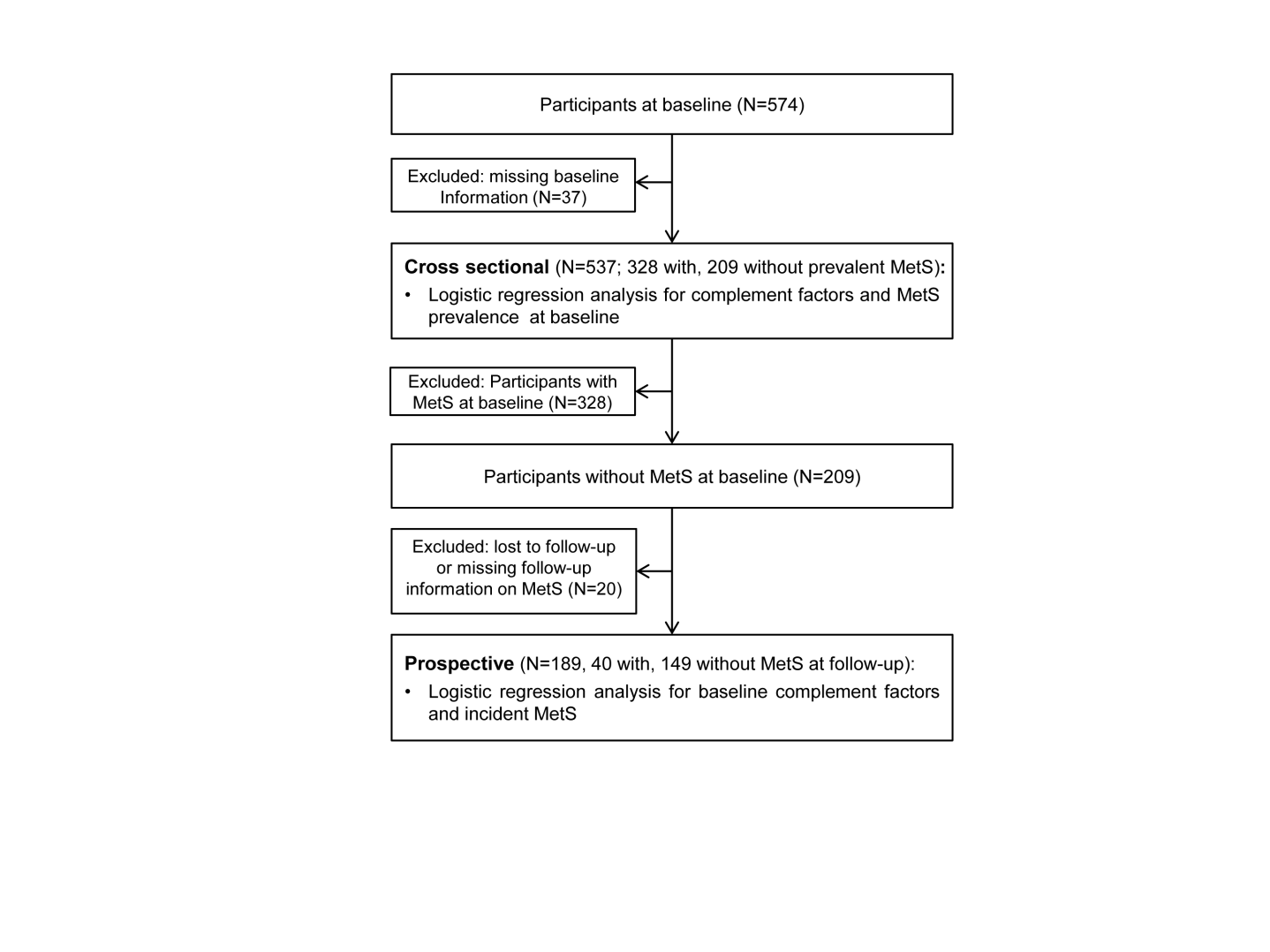


**Figure S2. Flowchart of the individuals included in the main analyses in this study.** **Abbreviations:** MetS, metabolic syndrome.


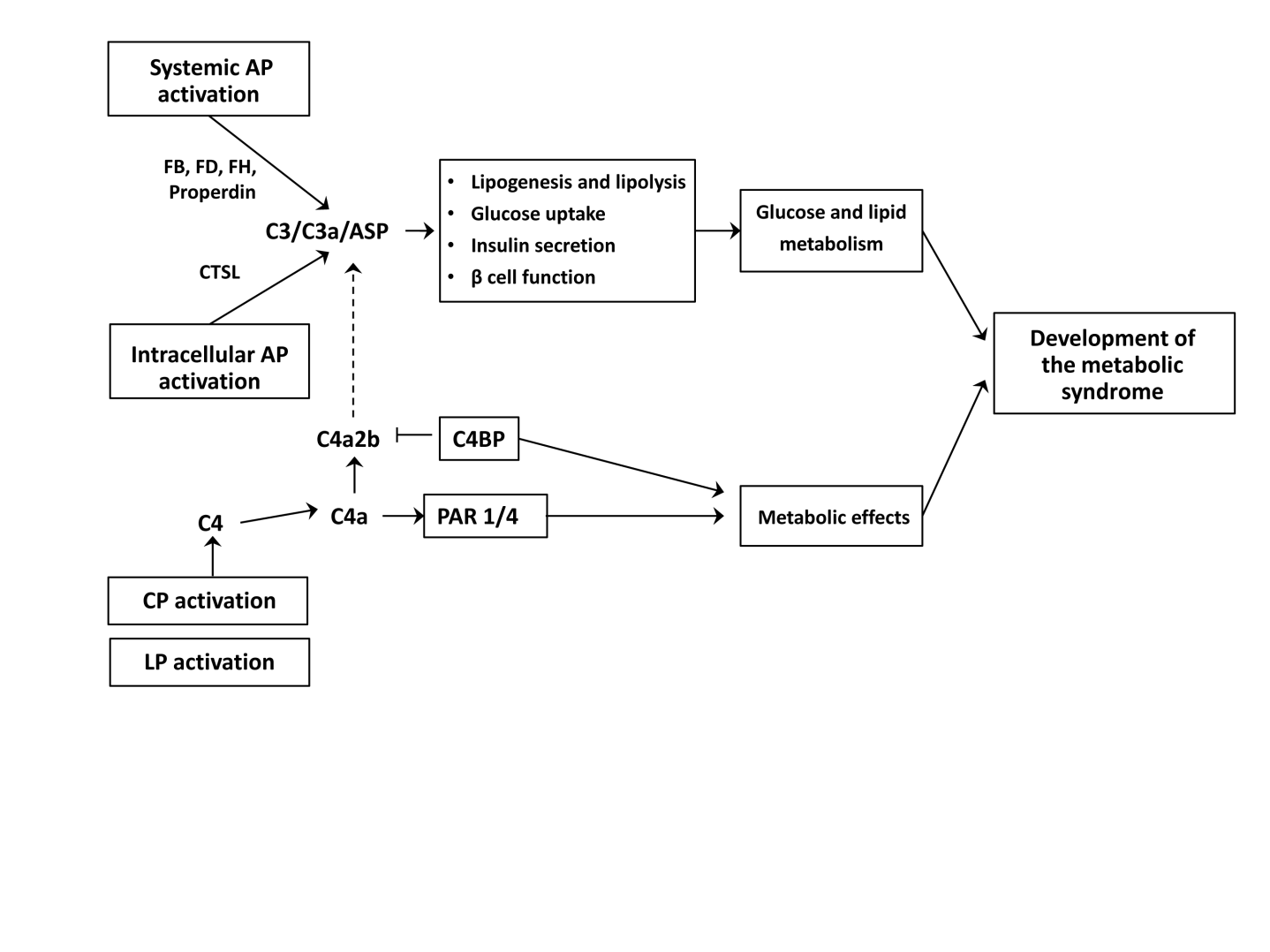


**Figure S3. Graphic summary of the possible explanations for the main findings.** **Abbreviations:** AP, alternative pathway; CP, classical pathway; TP, terminal pathway; FB, factor B; FD, factor D; FH, factor H ; ASP, Acylation stimulating protein; CTSL, cathepsin L; C4BP, C4 binding protein; PAR 1/4, protease-activated receptor 1 and 4.

**Table S1. Cross-sectional associations of complement with components of the metabolic syndrome (N=537)**

|  | **Waist (cm)** | **TG (mmol/L)^a^** | **HDL (mmol/L)** | **SP (mmHg)** | **DP (mmHg)** | **FPG (mmol/L)^a^** |
| --- | --- | --- | --- | --- | --- | --- |
|  | β [95% CI] | β [95% CI] | β [95% CI] | β [95% CI] | β [95% CI] | β [95% CI] |
| **C3 (SD)** | **5.47 [4.59; 6.35]** | **0.33 [0.27; 0.38]** | **-0.13 [-0.15; -0.10]** | **3.22 [1.71; 4.73]** | **1.96 [1.18; 2.73]** | **0.05 [0.03; 0.07]** |
| **C3a (SD)** | **1.94 [0.99; 2.89]** | 0.01 [-0.05; 0.07] | -0.02 [-0.04; 0.01] | **1.97 [0.50; 3.45]** | 0.15 [-0.62; 0.91] | 0.01 [-0.01; 0.03] |
| **Bb (SD)** | -0.53 [-1.51; 0.44] | **-0.09 [-0.15; -0.03]** | -0.01 [-0.04; 0.02] | -0.56 [-2.05;0.94] | **-0.92 [-1.69; -0.16]** | -0.02 [-0.04; 0.00] |
| **Factor D (SD)** | **2.06 [1.07; 3.04]** | **0.08 [0.02; 0.14]** | **-0.06 [-0.09; -0.03]** | -1.17 [-.70; 0.36] | -0.24 [-1.03; 0.55] | -0.02 [-0.04; 0.00] |
| **factor H (SD)** | **3.94 [3.03; 4.84]** | **0.15 [0.10; 0.21]** | **-0.08 [-0.11; -0.06]** | **1.68 [0.20; 3.16]** | **1.32 [0.56; 2.07]** | **0.04 [0.02; 0.06]** |
| **Properdin (SD)** | **2.01 [1.06; 2.95]** | **0.23 [0.18; 0.29]** | **-0.10 [-0.13; -0.07]** | **2.06 [0.60; 3.52]** | **1.50 [0.75; 2.25]** | **0.02 [0.00; 0.04]** |
| **C1q (SD)** | 0.21 [-0.74; 1.17] | **0.08 [0.02; 0.14]** | **-0.06 [-0.08; -0.03]** | 0.11 [-0.13; 1.57] | 0.26 [-0.49; 1.01] | **0.02 [0.00; 0.04]** |
| **C1-INH (SD)** | 0.57 [-0.41; 1.54] | 0.05 [-0.01; 0.11] | 0.01 [-0.01; 0.04] | 1.28 [-0.21; 2.77] | **0.90 [0.14; 1.67]** | 0.01 [-0.01; 0.03] |
| **C4 (SD)** | **2.30 [1.37; 3.24]** | **0.09 [0.03; 0.15]** | **-0.05 [-0.08; -0.02]** | **1.53 [0.07; 2.99]** | **0.92 [0.16; 0.67]** | -0.00 [-0.02; 0.02] |

**Abbreviations:** TG, triglycerides; HDL, HDL-cholesterol; SP, systolic blood pressure; DP, diastolic blood pressure; FPG, fasting plasma glucose.

^a^ TG and FPG were log_2_-transformed prior to the analyses.

All analyses were adjusted for age, sex, medication use (lipid-modifying, antihypertensive, and/or glucose-lowering), smoking status, alcohol consumption, physical activity, and energy intake.

**Table S2. Sensitivity analyses: Prospective associations of baseline concentrations of complement proteins, regulators and activated products with incident metabolic syndrome**

|  | **Outcome: incident metabolic syndrome** | | | |
| --- | --- | --- | --- | --- |
|  | Model 1 | | Model 2 | |
|  | OR 95% CI | *P* value | OR 95% CI | *P* value |
|  | *No use of lipid-modifying, antihypertensive, and/or glucose-lowering medication; N=97 ^a^, 12 incident cases (12%)* | | | |
| **C3 (SD)** | 1.27 [0.68; 2.38] | 0.460 | 1.22 [0.65; 2.31] | 0.538 |
| **C4 (SD)** | **2.63 [1.32; 5.23]** | **0.006** | **2.55 [1.27; 5.09]** | **0.008** |
|  | *No chronic or acute infections; N=183 ^b^, 39 incident cases (21%)* | | | |
| **C3 (SD)** | **1.63 [1.13; 2.34]** | **0.009** | **1.53 [1.05; 2.23]** | **0.029** |
| **C4 (SD)** | **2.05 [1.40; 3.02]** | **<0.001** | **2.09 [1.39; 3.15]** | **<0.001** |
|  | *No autoimmune disease; N=164 ^c^, 35 incident cases (21%)* | | | |
| **C3 (SD)** | **1.50 [1.03; 2.19]** | **0.034** | 1.36 [0.91; 2.01] | 0.132 |
| **C4 (SD)** | **1.98 [1.32; 2.99]** | **0.001** | **1.91 [1.24; 2.93]** | **0.003** |
|  | *No malignant disease and/or cancer; N=180 ^d^, 37 incident cases (21%)* | | | |
| **C3 (SD)** | **1.63 [1.13; 2.35]** | **0.009** | **1.55 [1.06; 2. 26]** | **0.024** |
| **C4 (SD)** | **2.07 [1.40; 3.06]** | **<0.001** | **2.07 [1. 37; 3.13]** | **0.001** |
|  | *No liver disease; N=186 ^e^, 38 incident cases (20%)* | | | |
| **C3 (SD)** | **1.62 [1.13; 2.32]** | **0.009** | **1.54 [1.06; 2.25]** | **0.025** |
| **C4 (SD)** | **1.99 [1.37; 2.91]** | **<0.001** | **2.05 [1.37; 3.08]** | **0.001** |

Model 1 (M1) is adjusted for age and sex.

Model 2: M1 + medication use (lipid-modifying, antihypertensive, and/or glucose-lowering, if applicable), smoking status, alcohol consumption, physical activity, and energy intake.

^a^ 92 individuals who used lipid-modifying, antihypertensive, and/or glucose-lowering medication at baseline and/or at follow-up were excluded.

^b^ 6 individuals with chronic or acute infections at baseline were excluded.

^c^ 25 individuals with a (suspected) history of autoimmune disease at baseline were excluded.

^d^ 9 individuals have malignant condition or cancer at baseline were excluded.

^e^ 3 individuals have liver disease at baseline were excluded.
